# Lupin Protein Isolate Structure Diversity in Frozen-Cast Foams: Effects of Transglutaminases and Edible Fats

**DOI:** 10.3390/molecules26061717

**Published:** 2021-03-19

**Authors:** Elaine Berger Ceresino, Eva Johansson, Hélia Harumi Sato, Tomás S. Plivelic, Stephen A. Hall, Jürgen Bez, Ramune Kuktaite

**Affiliations:** 1 Department of Plant Breeding, The Swedish University of Agricultural Sciences, Box 190, SE-234 22 Lomma, Sweden; eva.johansson@slu.se; 2 Department of Food Science, School of Food Engineering, University of Campinas, São Paulo, SP 13083-862, Brazil; heliah@unicamp.br; 3 MAX IV Laboratory, Lund University, Box 118, SE-221 00 Lund, Sweden; tomas.plivelic@maxiv.lu.se; 4 Department of Solid Mechanics, Lund University, Box 118, SE-221 00 Lund, Sweden; stephen.hall@solid.lth.se; 5 Fraunhofer Institute for Process Engineering and Packaging, Giggenhauser Str. 35, D-85354 Freising, Germany; juergen.bez@ivv.fraunhofer.de

**Keywords:** linoleic acid, glycerol, lecithin, healthy additives, food structure, structure-function relationship, food foams

## Abstract

This study addresses an innovative approach to generate aerated foods with appealing texture through the utilization of lupin protein isolate (LPI) in combination with edible fats. We show the impact of transglutaminases (TGs; SB6 and commercial), glycerol (Gly), soy lecithin (Lec) and linoleic acid (LA) on the micro- and nanostructure of health promoting solid foods created from LPI and fats blends. 3-D tomographic images of LPI with TG revealed that SB6 contributed to an exceptional bubble spatial organization. The inclusion of Gly and Lec decreased protein polymerization and also induced the formation of a porous layered material. LA promoted protein polymerization and formation of homogeneous thick layers in the LPI matrix. Thus, the LPI is a promising protein resource which when in blend with additives is able to create diverse food structures. Much focus has been placed on the great foamability of LPI and here we show the resulting microstructure of LPI foams, and how these were improved with addition of TGs. New food applications for LPI can arise with the addition of food grade dispersant Lec and essential fatty-acid LA, by improved puffiness, and their contributing as replacer of chemical leavening additives in gluten-free products.

## 1. Introduction

The use of lupin proteins as a substitute to soya proteins in industrial food applications have recently gained increased attention [1]. Proteins in the form of isolates or concentrates have been extracted from lupin flour in two stage processes using alkaline solution with isoelectric precipitation and salt with dilutive precipitation techniques [2]. Several extracting procedures have been proposed in order to maintain the native protein properties of lupin and make these proteins favorable to use in various food applications [3,4]. Lupin protein use as food ingredients in various food systems requires stable or as little modified as possible functional properties of protein in order to deliver desired functional properties of final food products [5]. In particular, emulsifying and foaming, are functional properties very attractive for food applications, allowing to deliver specific textures and structures of different food products. Thus, lupin has been successfully used as egg substitute in cakes, pancakes and bread [6,7,8], and as a functional ingredient in gluten-free products [9]. For example, lupin protein isolates can form heat-induced stable gels and a well cross-linked protein network that resist flow and retain structural shape under deformation [10]. During foaming, after protein unfolding protein aggregates are known to be formed through hydrophobic interactions and further strengthened by disulfide interactions. However, there is still a lack of understanding on the molecular mechanisms occurring at processing of lupin protein into protein-rich foods.

The demand for techno-functional properties of lupin proteins is of increasing interest, and here the enzymatic cross-linking of proteins using transglutaminase (TG, protein-glutamine γ-glutamyl-transferase E.C. 2.3.2.13) is commonly used to improve the techno-functional properties [11,12,13] and regarded as safe, although recent studies indicate concerns because the TG-treated wheat products can be immunoreactive patients with celiac disease [14]. Since TG catalyzes the acyl-transfer reaction between the γ-carboxylamide groups of protein (or peptide) bound glutamine and primary amines, the reaction outcome results into isopeptide bonded (inter- and intra- molecular) and cross-linked protein network. In addition, the reaction between the γ-carboxylamide groups and primary amines can be also used to improve nutritional value of lupin proteins. The cross-linking is of interest for improvement of techno-functional properties of foods, and this cross-linking depends greatly on the protein type [13] and the TG enzyme source used [5]. We have recently determined that the wheat gliadin solid foam was successfully cross-linked with high (vs. low) concentration of the TG enzyme from the unique strain *Streptomyces* sp. CBMAI 1617 [15,16]. In that system, the TG used, increased the foam bubble size and was responsible for rather homogeneous structure of the foams. Furthermore, linolenic acid was more favorable than glycerol in designing the specific structural morphologies of processed solid gliadin foams [16]. However, limited numbers of studies have been performed on lupin protein and it remains unclear how lupin protein cross-linking and formation of the protein network are affected by different edible additives. Therefore, the aim of this study was to investigate the impact of TGs and different edible fats on lupin structural-functional performance and polymerization. We also studied the effect of those components on lupin protein foam morphology at micro- and nanometric levels.

## 2. Results and Discussion

### 2.1. Effect of TGs and Additives on Lupin Protein Polymerization in Frozen-Cast Lupin Foams

**Figure 1:**
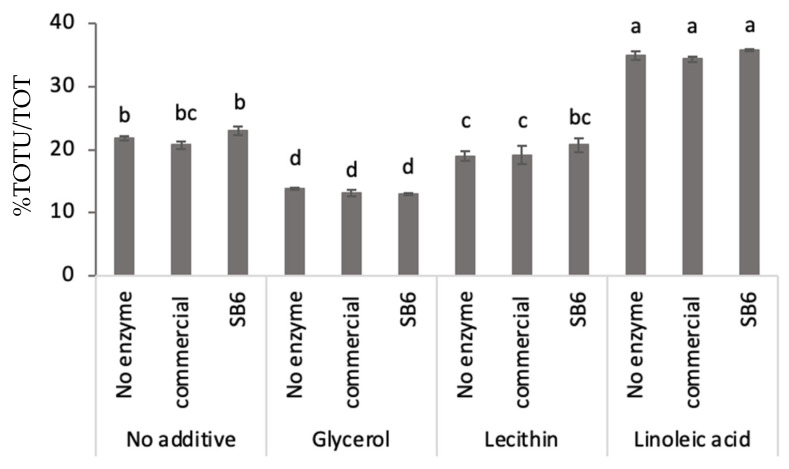
Protein polymerization measured as ratio of unextractable protein (TOTU) and total protein (extractable protein + unextractable protein) content (TOT), in solid lupin foams with and without different transglutaminases (commercial or SB6) and additives (glycerol, lecithin and linoleic acid). Error bars denote standard deviation; values with different letters (a–d) are significantly different; the means were compared at significant level of 5% using one-way ANOVA.

Lupin protein polymerization, calculated as the ratio between the unextractable protein fraction in the total amount of protein (including both unextractable and extractable protein fractions), differed significantly in the solid foam samples evaluated (Figure 1). Generally, limited effect on the protein polymerization was noted for the additions of TG, independent of TG source (commercial or SB6) used as compared to when no enzyme was added (Figure 1). Differently, addition of edible additives contributed to significant changes in the lupin protein polymerization. The addition of linoleic acid resulted in a significant increase in protein polymerization as compared to foams with no additive applied (Figure 1). Addition of glycerol and lecithin instead decreased the protein polymerization in lupin foams to lower levels than in the no additives samples, with the significantly largest decrease in the glycerol samples (Figure 1).

**Figure 2:**
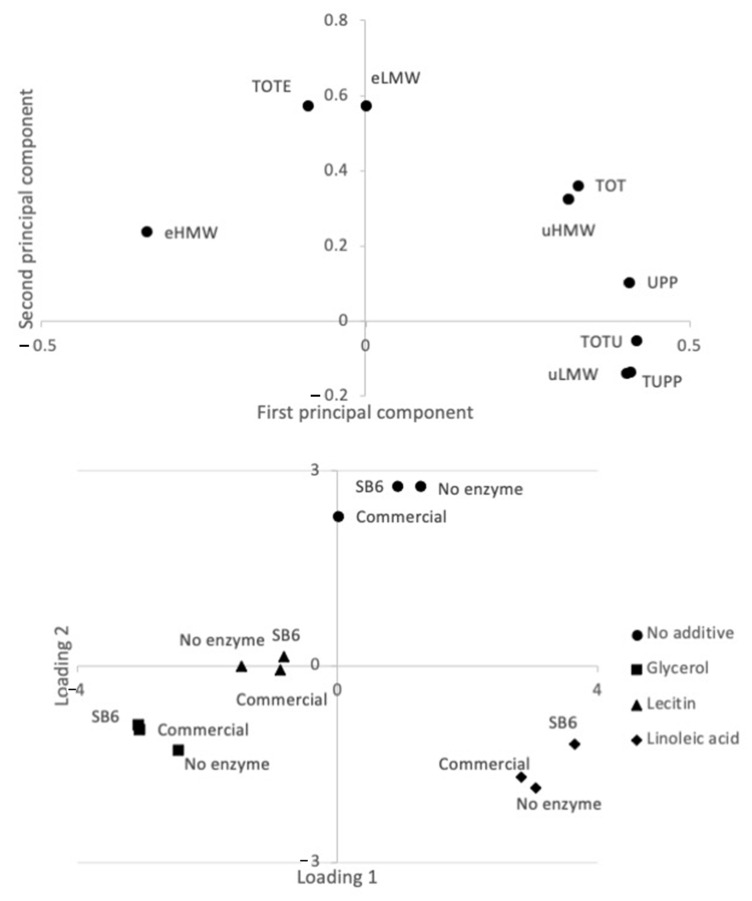
Score (**A**) and loading plot (**B**) from principal component analysis (PCA) of lupin foams: eHMW: SDS-extractable high molecular weight proteins; eLMW: SDS-extractable low molecular weight proteins; uHMW: SDS-unextractable high molecular weight proteins; uLMW: SDS-unextractable low molecular weight proteins; UPP: percentage of unextractable polymeric protein in total polymeric protein; TUPP: percentage of total unextractable protein; TOT: total protein; TOTU: total unextractable protein.

The fact that the addition of edible additives played a larger role for the lupin protein polymerization than the addition on TG was further elucidated by a principal component analysis (PCA) (Figure 2), clearly identifying four groups of samples based on edible additives used.

**Figure 3:**
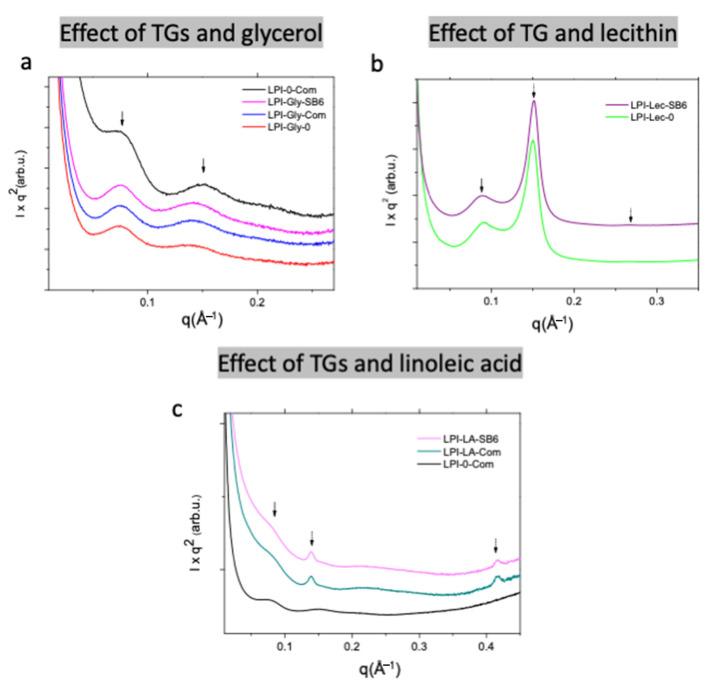
I × q^2^ vs. q Small Angle X-ray Scattering (SAXS) profiles of lupin foams. (**a**): effect of TGs and glycerol, (**b**): effect of TG (SB6) and lecithin and (**c**): effect of TGs and linoleic acid. Curves were vertically shifted for better visualisation.

Within the PCA, the first principal component explained 62% and the second principal component explained 32% of the variation. Protein factors connected to protein polymerization [17,18,19] were differenciated along the PC1, with those indicating increased protein polymerization showing positive values, while negative values were found for those indicating decreased polymerization (Figure 2a). Thus, corresponding with mean value comparisons (Figure 1), the glycerol and lecithin samples were found with negative PC1 values indicating the samples being less polymerized than the “no additive” and linoleic acid samples with positive PC1 values (Figure 2b). The decrease in protein polymerization by the addition of glycerol was further elucidated by the results obtained with Small Angle X-ray Scattering (SAXS) analysis (Figure 3a), identifying two broad scattering peaks corresponding to two interdomains d1 and d2, observed in all the samples containing glycerol, regardless of the presence of TGs (Figure 3, Table 1). For the sample without glycerol (LPI-0-commercial) smaller d1 and d2 values were observed in comparison to the other samples (Table 1), which indicate a differently organized system in the absence of glycerol. Glycerol with its three functional hydroxy groups per molecule is known to easily embedd and interact with the lupin protein matrix and connect to protein chains due to steric interactions [20,21], that plasticized and arranged the protein. Previous studies on gliadin have shown that the presence of glycerol drives the gliadins into a more organized state, less susceptible to polymerization [16].

The fact that differences were larger in samples with different additives than when TGs of different sources were used was verified also by the SAXS analysis. Especially, similar d-values were obtained for the LPI-Gly-commercial and LPI-Gly-SB6 samples, and for the LPI-LA-commercial and LPI-LA-SB6 samples, verifying similar effects of the two tested enzymes (Table 1).

Thus, our results suggested that, independent of TG source applied in the present study, addition of TG did not contribute to the cross-linking of the lupin proteins during lupin foam processing, even though previous studies have indicated an effect of TG on lupin protein polymerization [22]. Although the lack of protein polymerization of the lupin proteins with TG additions was not further evaluated in the present study, it was assumed that the high temperatures used in the spray-drying step for the production of lupin protein isolates (160–220 °C inlet) [3] may denaturate proteins and induce the formation of a pre-aggregated network, as found for wheat gluten [11] or self-assembled nanostructures as was observed in soy protein [23]. Industrial different types of processing of storage proteins is known to result in excessive aggregation [24].

Additionally, glycerol seemed to contribute negatively for lupin proteins to be rearranged in polymers under stirring in order to achieve a molecular or supramolecular interaction. First, it seemed that protein hydration was favored over the interaction between glycerol and LPI. Increased protein–water interaction was achieved, and the solvent structure propels non-polar residues of the protein to be buried inside the compact protein [25,26]. Secondly, studies have shown that, when in the presence of water, glycerol is mainly excluded from the protein domain, due to steric exclusion given its larger molecular dimension than water [27], which can contribute with protein folding into more compacted states, hindering novel protein interactions between the peptide chains [16]. Nevertheless, linoleic acid (LA) had a positive effect on promoting lupin protein polymerization, something that might be attributed to the improved mobility of the protein subunits by hydrophobic interaction between hydrophobic residues of the protein and LA. A study on blue lupin isolate, reported a high surface hydrophobicity of 2185 ± 67.0 A.U for this isolate [28], which is much higher than for the proteins of chick pea, faba bean, lentil and soybean [29]. The addition of linoleic acid creates an oil/water favorable interface or even emulsion where highly hydrophobic globular protein molecules, such as found in blue lupin [28] or soy [23], rapidly unfold [30]. It is extensively known that protein unfolding is a necessary step to promote novel interaction among amino acids for the creation of novel bonds [31]. Thus, our results indicated that linoleic acid together with the shear forces applied to rupture non-covalent interactions within the peptide chains created a suitable environment for the lupin proteins unfolding and subsequently refolding, as well as rearrangement with formation of new covalent and non-covalent bonds towards a state of mininum free energy at the interface [32].

To our knowledge, this is the first study to address the impact of lecithin on lupin protein crosslinking during foaming. Compared to the impact of Gly and LA, Lec had a very mild effect in reducing protein cross-linking among lupin proteins. Lupin proteins are rich in sulphydryl groups which are intramolecularly bonded and form a stable structure, while some of the free sulphydryl groups are present in the soluble small proteins of LPI [33]. Therefore, extensive rearragement of the disulfide bonds were limited, partially because the intramolecular bonds seemed to stabilize the structure, and because the hydrophilicity of lecithin did not promote the protein mobility in the same way as LA. The charged choline group in lecithin seemed in wheat doughs, to be of relevance for competing for the carboxilic group from proteins, and to slightly reduce the degree of crosslinking [34], which seems also to be the case for lupin proteins.

### 2.2. Lupin Foam Morphology and Structure as Affected by Additives

**Figure 4:**
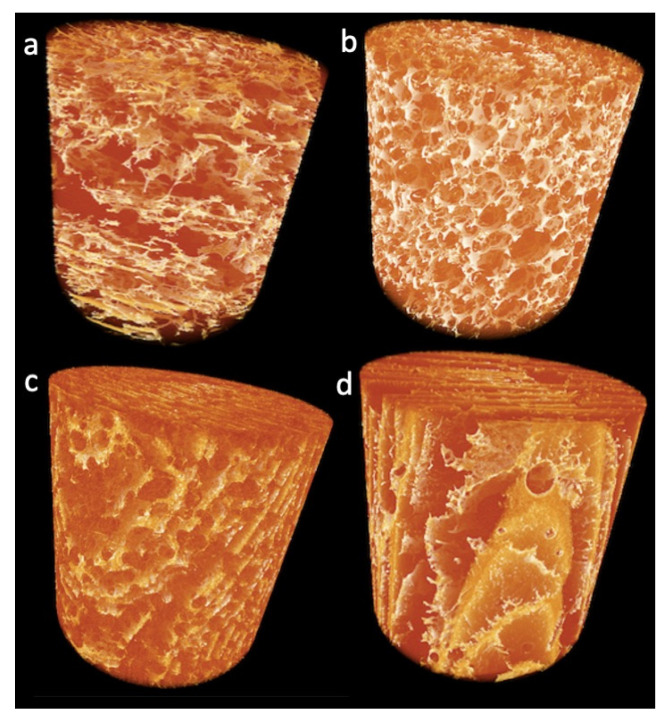
3D tomographs of lupin foams. (**a**): LPI-0-0; (**b**): LPI-0-SB6; (**c**): LPI-Lec-SB6; (**d**): LPI-LA-SB6.

**Figure 5:**
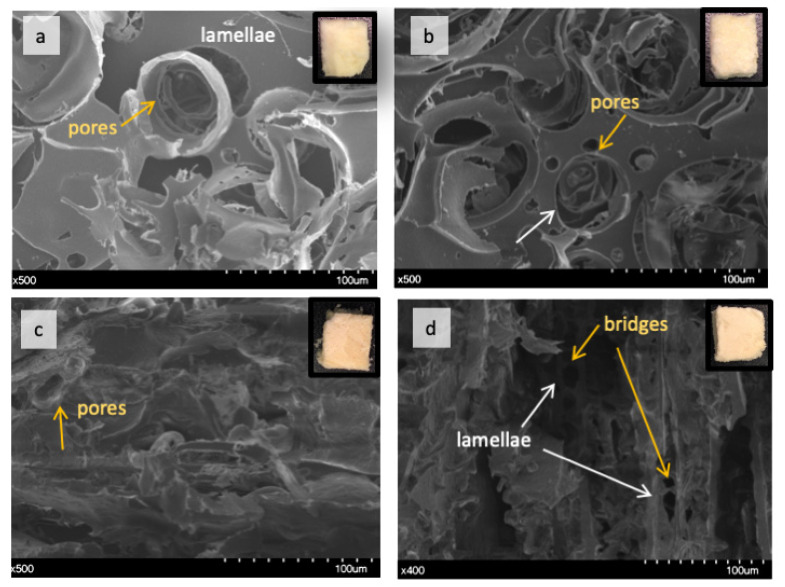
SEM micrographs of lupin foams (ca. 1 cm^3^). (**a**): LPI-0-0; (**b**): LPI-0-SB6; (**c**): LPI-LA-SB6; (**d**): LPI-Lec-SB6.

Globular proteins, such as the ones found in lupin, create structures which characteristics are driven by the interaction of the protein with water molecules and governed by other additives [35]. The impact of TG and food grade additives on the functionality of LPI varied largely in the present study as shown in the 3-D images of reconstructured foam from X-ray tomography (Figure 4; videos S1–S4; supplementary material) and SEM (Figure 5).

#### 2.2.1. Effect of TGs on Lupin Foam Morphology and Structure

**Figure 6:**
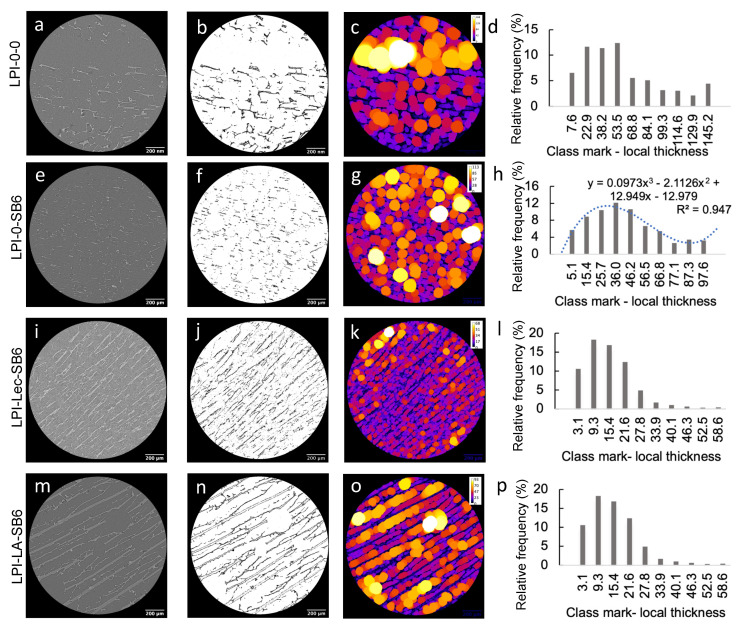
The images on the horizontal refers to the x-ray tomograph, tomograph image after threshold treatment, local thickness analysis and local thickness histogram. (**a**–**d**): LPI-0-0; (**e**–**h**): LPI-0-SB6; (**i**–**l**): LPI-Lec-SB6; (**m**–**p**): LPI-LA-SB6.

The morphology of lupin foam undergoes a significant improvement with the addition of SB6, compared to LPI-0-0 (Figure 4a, Video S1, supplementary material), in terms of governing the food foam design towards a mesoporous organized structure (Figure 4b, Video S2, supplementary material). For instance, bubble size distribution followed a polynomial function of third order given by the equation (Figure 6d, to the right). Bubble average size measured by means of local thickness (which represents the relative diameter of the largest sphere fitting inside an object) corresponded to ca. 36.41 ± 31.5 μm, in which 12% of the measured cells are having 36 μm of relative diameter. The well-organized foam lamellae stabilized bubbles had a defined circular shape as revealed by tomography (Figure 6e) and SEM images (Figure 5b). The arrows in Figure 4b highlight also the presence of pores with well defined borders of various diameters around the main bubbles. The fact that SB6 treatment of lupin proteins did not promote cross-linking to strength surface film was opposite to expected, but this result is similar to previously reported for zein protein networks [13]. Indeed, our produced lupin foams were rather fragile to handle. The contrasting results indicate that the mechanism related to the ability of TGs to improve foaming properties in LPI lies on the hydrolytic deamidation of the glutamine residues, where water is the acyl receptor in the absence of free lysine residues in sufficient amounts [16,36]. In fact, amino acid profile of the blue lupin protein isolate used in this study revealed an amount of 22.77 ± 2.76 g/100 g LPI of glutamic acid and 4.19 ± 0.51 g/100 g LPI of lysine [28]. Other studies had indicated, as well, that in the absense of enough lysine residues to be cross-linked with glutamine residues, deamination can be a prefered reaction by TGs [37]. The formation of charged groups resulting from deamidation increase the elestrostatic interaction between molecules in the interface and stabilizes the foam lamellar film [38] while eletrostatic repulsion by charged amino groups prevents interfacial self-assembly [39].

Comparatively, LPI-0-0, i.e., with no addition of SB6, presented a less organized structure (Figure 3a, Video S1, supplementary material), but with evidences of a more compact foam lamellae, which is seen in Figure 5b, thresholded image, and even more clearly seen in SEM images (in Figure 5a as indicated). The cellular structure of presented cells were more randomized in size which did not follow a polynomial regression and were on average 48.11 ± 46.37 μm.

Various factors are involved in foamibility of lupin proteins; protein diffusion to the air-water interface, followed by unfolding on the surface and ability to form a film or lamellae are key-factors driven by protein intrinsic properties [40], which are improved by the addition of TGs [16,37]. Various studies report excellent foaming properties and foam stability for white and blue lupin proteins when measured by increased volume, e.g., >70% by Vogelsang-O’Dwayer [28] and 89% by Piornos et al. [41] at relative low concentrations such as ca. 3 and 2%, respectively. Even though, protein concentration in this study was as high as 18%, and the methods for measuring foamability of lupin protein isolates are variable, there is good evidence that lupin protein isolate can be largely applied for the manufacturing of aerated foods, not to mention that lupin proteins stands out among other pulses for their bioactivity potential [42]. It is also clear from this study, that the addition of TGs could modulate and increase the foam quality, which represents a significant advance to increased control and predictability of the LPI behavior in desired structures of interest for food applications and possibly bioactive compounds delivery.

#### 2.2.2. Effect of Lecithin and Glycerol on Lupin Functional Structuring Performance in Frozen-Cast Foams

In this study, the addition of lecithin and glycerol resulted in less polymerized proteins, especially for glycerol. The fact the glycerol LPI foams desintegrated during handling for image analysis suggested a lack of disulfide bonds among lupin proteins when treated with glycerol which had a detrimental effect on the material integrity. The same was not observed for lupin treated with lecithin, indicating that this sample (LPI-Lec-SB6) had enough protein bonds of relevance to maintain the shape of the produced foam [16].

The effect of lecithin can be better understood by examining the tomography images and video (Figure 4c, Figure 6i,j, Video S3, supplementary material) which reveals macroporous aligned layers with up to 0.6 mm in length. SEM image of the core matrix (Figure 5d) shows that the pores in the layers are set apart by protein briges. Local thickness analysis (Figure 6k,l) further revealed that these layers were thinly separated (Figure 6k,l) by approximately 17 μm, with a distance variation in the range of 3.09–27.77 μm. Pores in the matrix, represented by the brightest color (Figure 6j), were found to measure between 52 and 58 μm in relative diameter (~0.7%), suggesting that the pore size was comparable to the pore sizes found in LPI-0-0 (ca. 12% of the bubbles in LPI-0-0) having approximately 53.5 μm in diameter, but bigger than average bubble diameter in LPI-0-SB6.

From a porous foam matrix as found in LPI-0-0 and LPI-0-SB6, the addition of lecithin imparted structural evolution to aligned macroporous layers in LPI-Lec-SB6. The investigation of the interaction between soy lecithin and soy globulins by Li, Li and Guo [43] revealed that hydrophobic forces promoted a binding between those two components changing surface activity of the proteins, which might have been the case for LPI and lecithin. The binding of Lec to protein induced the protein unfolding and increase the exposition of negatively charged residues, however, lecithin can aggregate at the surface in concentractions over 3% [44], which could have displaced LPI molecules towards the most hydrophobic phase. On the other hand, in the formation of these layers, an eletrostatic mechanism of stabilization might be involved as SB6 deamidation of glutamine residues lead the formation of charged groups in water that are able to stabilize the protein layer and prevent self-assembly along negatively charged phosphate groups (PO^3−^), similarly as was was observed in soy lecithin [45].

SAXS analysis on the impact of soy lecithin into the nanometric foam matrix showed that the interdomain distances d1 significantly decreased compared to the samples without lecithin (see Figure 3b and Table 1). The values for LPI-Lec-0 and LPI-Lec-SB6 were 68.5 and 70 Å, respectively, at least 10 Å lower than d1-values for the other samples. The presence of SB6 and the LPI-Lec foam, slightly increased the d1 value. Peak shifting values due to the presence of SB6 were reported in our previous studies [16]. Interestingly, on the lecithin modified LPI samples, two new peaks were observed at higher q-values. The first one, namely d_1,H_ on Table 1, was sharper and even more intense than the d1-peaks. The second one, d_2,H_ (Table 1), was weaker and seemed to be a second order reflection of the same structure. It is also important to notice that the relation between peak positions was 1:√3, characteristic of a hexagonal organization [46,47]. The formation of hexagonal structures by lecithins has been widely reported and could be favoured in the presence of other non-polar compounds [48], which could be related in this study to the intereaction with LPI.

These findings indicated that a combination of eletrostatic repulsive forces, lecithing hydrophilicity (each phospholipid can imbibe 6 to 10 water molecules [49]) and strong hydrophobic interactions were likely driving the design of LPI-Lec-SB6 thinly separated lecithin-lupin protein layers. Other studies have shown the effect of lecithin in preventing casein micelle aggregation in the presence of whey proteins, suggesting lecithin to be an effective dispersing and separating agent of proteins [50].

Glycerol presented a weakning effect on lupin foaming, probably associated with its effect on decreasing polymerization. As foam samples were very brittle and unstable to withstand tomography and SEM analysis, glycerol effect will be further discussed only in light of SE-HPLC and SAXS experimental results. The d_1_ and d_2_ values (characteristic distances) observed by SAXS for LPI-Gly-0 rtyt 84.5 Å and 44.8 Å. The presence of “commercial” TG slightly shifted these distances to lower values, which ertr 83 Å and 44.5 Å for d1 and d2 respectively. Higher correlation distances in gliadin/glycerol mixtures were associated with increased polymerization [16,51]. In this study for LPI, the distances decreasef, indicating in fact a less polymerized matrix.

#### 2.2.3. Effect of Linoleic Acid on Lupin Structuring Performance in Frozen-Cast Foams

Linoleic acid was the additive presenting not only a major impact in protein polymerization, as well as in driving the formation of solid lamellar structures in foamed lupin. Tomographic images (Figure 6m,n) show formed layers with length varying between 0.6–1.2 mm, as well as the presence of isolated bubbles embedded in the foam structure with diameters up to 262 μm. Layer thickness, as shown by local thickness analysis, measured up to 20 μm (Comparative Figure 5o,p). The distance between the dark-colored layers could be as large as 80 μm. During freezing time, this well developed protein film separated from the water, forming the various layers, and even stabilized some bubbles. This was different from previous studies with oat dough liquor, showed that the surface of foams when impregnated with lipids results in lower surface tension [52] an in the absence of fatty acids, globulins from faba beans were found to strongly interact to each other through hydrophobic interactions of disulfide bonds, forming spherical protein particles [53].

SAXS results (Figure 3c) also showed a clear distinguished structure in comparison to the samples without linoleic acid. New sharper peaks, named as d_1,L_ and d_3,L_ and indicated with dotted arrows, were observed for LPI-LA-commercial and LPI-LA-SB6. The difference on the d-values for the different TGs are not significant (Table 1). The relation on the Bragg peak position d_1,L_:d_3,L_ was 1:3 which could be correlated to the presence of a lamellar structure. The absence of the second order reflection could be due to the composition of the sample, but further needs to be investigated in order to confirm this assumption. LPI/lipids/water systems could form protein–lipid complexes composed a fatty acid compartment and proteinaceous shell. This complex formation occurred as a result of partial protein development and hydrophobic binding between protein and fatty acid [54], or a similar mechanism as observed in lipid-based carriers [55].

The results given above suggest that the hydrophobic interaction between lupin proteins favored developing of a stable cross-linked matrix and rather thick matrix walls as shown in Figure 5c through protein bond rupture and rearragements that favored the formation of a relatively continous surface. The fact that LA and LPI are compatible in forming stable layers is of great relevance and could find many applications in the confectionary industry, or even as the carrier of hydrophobic bioactives substances. Studies show that consumers are not only demanding snacks which are free from specific ingredients such as carbohydrates and trans fatty acids, but require snacks which are rich in protein and healthy fats, such as “keto vegan snacks” [56].

## 3. Materials and Methods

### 3.1. Lupin Protein Isolate

Agglomerated lupin protein isolate (LPI) was produced at ProLupin (Grimmen Germany) from the seeds of blue lupin (*Lupinus angustifolius* cv. Boregine) and kindly provided by Fraunhofer IVV (Freising, Germany). LPI was produced from blue lupin with amounts of protein 92.6% (N × 6.25), fat 0.92%, ash 5.52% and dietary fiber < 0.1% that were characterized in the similar material [28].

### 3.2. Additives

Two types of transglutaminase (TG), a commercially available preparation kindly provided by Shangai Kinry Food Ingredients Co. (Kuala Lumpur, Malaysia) (designated here and onwards as “commercial”) and newly sourced bacterial TG preparation, SB6 [15] were tested. Soya lecithin granules (Lec) (Solgar, Lynbrook, NY, USA), glycerol (Gly) (99.5%; Karlshams Tefac AB, Karlshamn, Sweden) and linoleic acid (LA) (technical grade, Sigma Aldrich, Deisenhofen, Germany) were used as additives. The samples resulting from the combination of additives and the TGs are described in Table 2.

### 3.3. Foam Preparation

In a conical tube of 50 mL containing 15 mL of water, lupin (2.7 g), TGs (4.68 U/g of lupin) and additives (0.3 g/g of lupin) were mixed using an Ultra-Turrax blender (TP18/10 with dispersing tool S25-18G, Janke and Kunkel Gmbh and Co., Staufen, Germany) during 45 s. Concentration of each ingredient was chosen based on previous studies [16]. After mixing, the liquid foams were rapidly transferred to 60 × 15 mm petri dishes and frozen at −80 °C. Afterwards, the frozen foams were freeze-dried (Cool Safe ™, Scanvae, Denmark) over 48 h, and kept over silica gel in a desiccator prior to the analyses. For SE-HPLC analysis, samples were milled into powder with an analytical mill (IKA A10, IKA-Werke, Staufen, Germany) and stored at −20 °C until further analysis.

### 3.4. Chemicals

A Milli-Q purified water (Millipore Corporation, Billerica, MA, USA) was used in all experimental procedures and during wet foam-making. The following chemicals of analytical grade were used during the analysis: sodium dodecyl sulfate (SDS) was obtained from Duchefa Biochemie (Haalen, the Netherlands), NaH_2_PO_4_ and trifluoroacetic acid (TFA) were purchased from Merck (Darmstadt, Germany) and acetonitrile was acquired from VWR BDH Prolabo (HPLC grade,VWR Chemicals, Stockholm, Sweden).

### 3.5. Size Exclusion-High Performance Liquid Chromatography (SE-HPLC)

Lupin protein extraction from the matrix was performed from the material as follows: dried foams were finely disintegrated in a mortar with the aid of a pestle and 16.5 mg of each sample was weighed in a centrifuge tube, subsequently filled with 1.4 mL of buffer (0.5% w/v SDS 0.05 M NaH_2_PO_4_, pH 6.9). First, the extractable proteins were obtained by vortexing the samples for 10 s at maximum speed (Whirli VIB 2, Labassco, Sweden), followed by vigorous shaking at 2000 rpm (IKA-VIBRAX VXR, Ika-Labortechnik, Staufen, Germany) and centrifugation at 15,000 g during 30 min (Sorvall Legend micro 17, Thermo Scientific, Fairlawn, NJ, USA). The supernatant was collected in HPLC vials for subsequently protein separation, while 1.4 mL of buffer was added to the tube containing the pellet. The insoluble proteins in the pellet were extracted through sonication using an amplitude of 5 mm during 45 s (Sanyo Soniprep, Tamro, Sweden). Extractions were performed in triplicate. HPLC were performed using a Water 996 Photodiode Array Detector (Waters, Wilmington, MA, USA, USA) equipped with a size exclusion column (Biosep-SEC-S 4000, Phenomenex, Torrance, CA, USA) at room temperature. For the analysis, 20 mL of sample was injected onto the column and eluted by an isocratic flow of 0.2 mL/min (50% acetonitrile, 0.1% TFA; 50% H_2_O, 0.1% TFA). Chromatograms were extracted at 210 nm (Empower Pro Waters, Water Corporation, Milford, MA, USA). The SE-HPLC chromatograms of SDS-extractable and SDS-unextractable protein were arbitrarily divided into two intervals, from 8.1–11.5 min and 11.5–26 min, corresponding to high molecular weight (HMW) and low molecular weight protein (LMW), respectively. The total unextractable protein was calculated as the sum of unextractable HMW and LMW and used to calculate the ratio of unextractable total protein and relative total amount of protein in the samples (TOT).

### 3.6. Tomography

The acquisition of 3D volume images was performed using a Zeiss XRadia XRM520 at the 4D Imaging Lab, Lund University, Sweden. A polychromatic cone beam with a source voltage of 40 kV, a power of 3 W and exposure time of 4 s was obtained by an X-ray source. To compose 3-D rotating images, 1993 radiographs with width and height of 1014 pixels were acquired over 360° with an optical magnification factor = 4 and the cubic voxel dimension was 1.5014 µm. The Fiji software (https://imagej.net/Fiji) was used to perform local thickness analysis [57] in one slide of each sample that illustrates the foam internal structure dimension as follows: slide number 682; 696; 793; 609 of the samples LPI-0-0, LPI-0-SB6; LPI-Lec-SB6; LPI-0-LA, respectively. Prior to local thickness analysis, micrographs were transformed by adjusting the threshold ratio to best delineate foam internal structure and images were calibrated according to the magnification of the image. After local thickness analysis, histogram data was collected and transformed into relative frequency percentage for comparative analysis between the samples.

### 3.7. Scanning Electron Microscopy (SEM)

The morphology of lupin foams was revealed using a Scanning Electron Microscope (Hitachi SU3500, Tokyo, Japan) at the Department of Biology, Lund University, Sweden. Prior to analysis samples were kept in a desiccator containing silica-gel until the analysis, then transversally cut with a scalpel and air-blowed to remove small sample fragments scatted during cutting. Afterwards, the samples were fixed onto aluminum holders with a double-sided bond tape and immediately sputter-coated with gold for 55 s using a Cressington Sputter Coater 108 auto (Cressington Scientific Instruments, Watford, UK).

### 3.8. Small Angle X-ray Scattering (SAXS)

The SAXS experiments were performed at the beamline 1911-4, MAX IV Synchroton Laboratory in Lund, Sweden. The data were recorded using a bi-dimensional hybrid pixel X-ray detector (Pilatus 4531M, Dectris, Baden, Switzerland placed at a distance of 1911.36 mm from the sample. The scattering vector q varied in the range of 0.01 and 0.5 Å, where q is defined as q = (4πλ)sinθ; 2θ is the scattering angle and λ = 0.91 Å is the incident wavelength. The data were reduced using the software bli9114 [58]. The SAXS curves were normalized by the integrated intensity incident on the sample during exposure time and corrected by sample absorption and background.

### 3.9. Statistical Analysis

To evaluate differences in the proportion of unextractable protein, analysis of variance was performed followed by calculation of means with significances differentiated by the use of Tukey post hoc analysis (*p* < 0.05), using the Minitab express version 1.5.1. The Statistical Analysis System (v.8, SAS Institute, Cary, NC, USA) program was used for principal component analysis (PCA). The relative frequency distribution of local thickness data was performed using the software Microsoft Excel for Mac version 15.28.

## 4. Conclusions

LPI prepared at standard temperatures through spray-drying are highly cross-linked due to the production process and therefore, transglutaminases have limited influences on further cross-linking of the proteins. However, the TGs, specifically the recently sourced SB6, contribute to improvements in terms of an ordered and well-developed protein foam. Furthermore, additions of lecithin or linoleic acid contribute and lead to morphological and nanostructure evolution of the samples. While SB6 significantly contributes to the foam homogeneous periodic morphology despite not contributing to cross-links, lecithin promoted the formation of new hexagonal structures at the nanometric scale of the foam matrix whereas linoleic acid shows the existence of lamellar structures or complex protein–lipid bonding at the nanometric and molecular range of the foam walls. The highest increase in polymerization of LPI was obtained by addition of linoleic acid, probably as a consequence of the interaction of linoleic acid with hydrophobic residues of LPI.

Utilization of LPI, as an interesting healthy ingredient, and in combination with the additives lecithin and LA, which are also health promoting ingredients, is a suitable option and results in the formation of thinly separated layers which can impart texture-appeal to food products. However, if the purpose is to obtain a homogenous foam, the addition of lecithin and linoleic acid is discouraged, but the addition of TG can be highly beneficial.

## Figures and Tables

**Table 1:** Characteristic distances, d-values in Å, observed by SAXS.

Samples	Distances (Å) *					
	d_1_	d_2_	d_1,H_	d_2,H_	d_1,L_	d_3,L_
LPI-0-commercial	81.6	41.2	-	-	-	-
LPI-Gly-0	84.2	44.2	-	-	-	-
LPI-Gly-commercial	82.3	43.6	-	-	-	-
LPI-Gly-SB6	82.7	43.5	-	-	-	-
LPI-Lec-0	68.5	-	41.8	23.6	-	-
LPI-Lec-SB6	70.0	-	41.4	23.5	-	-
LPI-LA-commercial	83.4	-	-	-	45.1	15.1
LPI-LA-SB6	83.4	-	-	-	45.0	15.0

* peak distances not detectable were marked with “-“.

**Table 2:** Composition of solid lupin foams consisting of diverse additives mixtures.

Samples	Lupin Content ^a^		TG ^b^		Gly Content ^c^		Lec Content ^d^		Linoleic Acid Content ^e^		Water
	g	wt%	mg	U/g	g	wt%	g	wt%	g	wt%	mL
LPI-0-0	2.7	18	0				0				15
LPI-0-commercial	2.7	18	40	4.68			0				15
LPI-0-SB6	2.7	18	10	4.68			0				15
LPI-Gly-0	2.7	18	0		0.81	30					15
LPI-Gly-commercial	2.7	18	40	4.68	0.81	30					15
LPI-Gly-SB6	2.7	18	10	4.68	0.81	30					15
LPI-Lec-0	2.7	18	0				0.81	30			15
LPI-Lec-commercial	2.7	18	40	4.68			0.81	30			15
LPI-Lec-SB6	2.7	18	10	4.68			0.81	30			15
LPI-LA-0	2.7	18	0						0.81	30	15
LPI-LA-commercial	2.7	18	40	4.68					0.81	30	15
LPI-LA-SB6	2.7	18	10	4.68					0.81	30	15

^a^ LPI content in weight (g) and weight percent (wt%) of the water and gliadin mixture. ^b^ TG content in weight (mg) and concentration of TG in the lupin/TG mixture. ^c^ Glycerol content in weight (g) and weight percent (wt%) of the lupin/glycerol mixture. ^d^ Lecithin content in weight (g) and weight percent (wt%) of the lupin/linoleic acid mixture. ^e^ Linoleic acid content in weight (g) and weight percent (wt%) of the lupin/linoleic acid mixture.

## Data Availability

Not applicable.

## Supplementary Materials

The following are available online, Video S1–S4. Model reconstruction of lupin foams based on X-ray tomography images; S1: LPI-0-0; S2: LPI-0-SB6; S3: LPI-Lec-SB6; S4: LPI-LA-SB6.

## Sample Availability

Samples of the compounds are not available from the authors.
